# Effect of Dietary *Boswellia serrata* Resin on Growth Performance, Blood Biochemistry, and Cecal Microbiota of Growing Rabbits

**DOI:** 10.3389/fvets.2019.00471

**Published:** 2019-12-20

**Authors:** Ismail E. Ismail, Sameh A. Abdelnour, Sabry A. Shehata, Mohamed E. Abd El-Hack, Mohamed A. El-Edel, Ayman E. Taha, Michele Schiavitto, Vincenzo Tufarelli

**Affiliations:** ^1^Department of Poultry, Faculty of Agriculture, Zagazig University, Zagazig, Egypt; ^2^Department of Animal Production, Faculty of Agriculture, Zagazig University, Zagazig, Egypt; ^3^Department of Animal Husbandry and Animal Wealth Development, Faculty of Veterinary Medicine, Damanhour University, Damanhour, Egypt; ^4^Department of Animal Husbandry and Animal Wealth Development, Faculty of Veterinary Medicine, Alexandria University, Rasheed, Egypt; ^5^Italian Rabbit Breeders Association (ANCI-AIA), Volturara Appula, Foggia, Italy; ^6^Department of DETO, Section of Veterinary Science and Animal Production, University of Bari Aldo Moro, Bari, Italy

**Keywords:** *Boswellia serrata*, growth, immunity, anti-oxidant, intestinal microbiota, rabbits

## Abstract

The present study aimed to examine the impact of dietary supplementation of *Boswellia serrata* (BS) (frankincense) resin on growth and carcass traits, blood hematology, serum metabolites and cecal microbiota of growing rabbits. One hundred New Zealand White (NZW) growing male rabbits (6-weeks old) were divided randomly into five groups using different levels of BS (0.00, 0.25, 0.50, 0.75, 1.00 g/kg diet, respectively). When compared to the control diet, daily body weight gain (BWG) and feed conversion ratio (FCR) of rabbits fed BS enriched-diets were improved, while feed intake was significantly decreased. A gradual depression (P < 0.01) in serum triglycerides (TG), total cholesterol (TC) and low density lipoproteins (LDL) were observed with increasing BS level the in diet. Total bacteria count, *E. coli* and salmonella populations were lower (*P* < 0.05) in rabbit groups fed diet enriched with BS than that of the control group. Based on these findings, the dietary supplementation of *Boswellia serrata* enhanced growth, feed efficiency, anti-oxidant status, and minimize cecal pathogenic bacteria in rabbits.

## Introduction

Natural growth enhancers are a broadly used approach to improve the utilization of nutrients after the ban on anti-biotics in rabbit diets (1). In the rabbit industry, phytogenic dietary additives are products derived from plants used in rabbit feeding to promote their productive performance (1, 2). Supplying animals and poultry with a diet enriched with phytogenic feed additives may have beneficial effects such as improvement of nutrient utilization, gut health and stimulation of immune system (1–3). Some of the remedial features of herbal plants and/or their active molecules are due to their secondary metabolites effect (3, 4).

*Boswellia serrata* (BS) or frankincense from the Arabian Peninsula belonging to the *Burseraceae* family. It possesses various therapeutic activities, for example, bactericidal and anti-inflammatory properties, due to the precence of many aromatic compounds with boswellic acid the most active molecule (5–9). Mono and diterpenes, ethyl acetate, octyl acetate, and methylanisole are natural anti-oxidants that constitute the major components of BS (5, 6). Application of the BS as a phytogenic in broiler diet can increase productive performance and enhance quality of meat (5, 6, 8, 9). Al-Yasiry et al. (8) reported that broiler chickens that were fed diets containing different levels of BS (0, 3, 4, or 5%) have increased significantly in their body weight, feed efficiency and absorption thereby, enhanced growth performance. Moreover, the broilers meat produced had lower fat content by diets supplemented with 3 or 4% BS (8, 9). Interestingly, it has been recognized that BS has enormous benefits as a natural product: it does not cause any residue in animal meat, as well as increases globulin, lymphocytes and improves the animal immune response (9). This suggests that BS is a natural favorable product especially in tropical and subtropical regions when there is a threat to the immune system from pathogens to reduce the massive use of anti-biotics (8, 9). These therapeutic activities of BS can perhaps be used in livestock species to obtain many positive effects on animal production and well-being (8). There is a lack of studies dealt with BS on rabbit performance. Therefore, the current study indicates an assessment of the impact of BS as a natural supplement on growth and carcass parameters, blood traits, and intestinal microbiota in rabbits.

## Materials and Methods

The present research was conducted in the Rabbit Research Unit, Department of Animal Production, Faculty of Agriculture, Zagazig University, Egypt. All procedures of this experiment were performed according to the local Experimental Animal Care Committee and ethics were approved by the Institutional Committee. Animals were taken care of to use the husbandry instructions derived from the standard operating procedures of Zagazig University.

### Animals and the Experimental Design

One hundred weaned males New Zealand White growing rabbits (mean body weight 778 ± 17 g) at 6 weeks of age were used in this experiment. Animals were gently acquired from Faculty of Agriculture, Zagazig University (Rabbit Research Unit). The random division of fryers into five equal groups was done (20 rabbits per group). The study lasted for 6 weeks and ended at 12 weeks of age. The dietary treatments tested in the experiment were as follows: C, control (basal-diet without feed additive), BS0.25, basal-diet + 0.25 g/kg diet of *Boswellia serrata* (BS), BS0.5, basal-diet + 0.50 g BS/kg diet, BS0.75, basal-diet + 0.75 g BS/kg diet and BS1.0, basal-diet + 1.0 g BS/kg diet).

Animals were fed to meet their nutritional requirements as reported by De Blas and Mateos (10). The ingredients and diet analyses, in pelleted form, were performed according to AOAC (11) as in Table 1. Animals were reared in stainless steel cages (45 cm high × 50 cm long × 35 cm wide) having automatic nipple drinkers and feeders. Each treatment had five cages (four rabbits each). Rabbits were housed in clean and well-ventilated room; the temperature average is about 18°C and 70% relative humidity. White led light (1fc) was provided for 14 h daily. Rabbits were fed *ad libitum* during the feeding experimental period. *B. serrata* was purchased from a local market, Zagazig City, Sharkia, Egypt, and then ground and added to diet before pelleted. The BS used in the experiment is composed by essential oil (4–10%), pure resin (65–85%), and mucopolysaccarides (21–22%), containing pentacyclic triterpene acids and tetracyclic, in addition to other active components like 3-O-acetyl-11-keto-β-boswellic acid (AKBA) and 11-keto-β-boswellic acid (KBA).

**Table 1:** Composition of the basal diet of growing rabbits (as fed).

**Items**	**Basal diet**
**Ingredient, %**	
Soybean meal (44% crude protein)	20.0
Yellow corn	20.0
Barley grain	10.0
Wheat bran	16.0
Berseem hay	30.0
Molasses	2.0
Premix^*^	0.5
Salt	0.5
Limestone	1.0
**Calculated composition, %**	
Crude protein	17.50
Metabolizable energy, MJ/kg	7.95
Phosphorus (Available)	0.20
Calcium	0.88
**Analyzed composition (%, on dry matter basis)**	
Organic matter	90.57
Dry matter	88.06
Crude fiber	12.33
Crude protein	16.54
Ether extract	2.25
Nitrogen-free extract	59.45
Ash	9.43

* *Each kg of premix (minerals and vitamins) contained: vit. A, 20,000 IU; vit. D3, 15,000 IU; vit. E, 8.33 g; vit. K, 0.33 g; vit. B1, 0.33 g; vit. B2, 1.0 g; vit. B6, 0.33 g; vit. B5, 8.33 g; vit. B12, 1.7 mg; pantothenic acid, 3.33 g; biotin, 33 mg; folic acid, 0.83 g; choline chloride, 200 g*.

### Growth Performance

Feed intake (FI), live body weight (LBW), body weight gain (BWG), and feed conversion ratio (FCR) were assessed according to the methodology described by Abdelnour et al. (1). Rabbits biweekly were weighed throughout the experimental period to determine both LBW and BWG. The amount of feed consumed was calculated biweekly, by weighing the remaining amounts of feed, and then subtracting it from the initial amount before presenting the new quantity.

### Carcass Measurements

At 12 weeks of age, five rabbits were randomly selected from each group, fasted for 6 h, weighed individually and according to Islamic procedures the rabbits were slaughtered, and after complete bleeding, the subjects were skinned, viscera removed, and then carcass weighed (3). The carcass percentage (hot eviscerated carcass weight of including head, heart, liver, and abdominal fat) was measured by dividing the weight of the carcass on the pre-slaughter weight and expressed as a percentage (12). Internal organs of rabbit sampled were expressed as relative weights to the pre-slaughter weights of rabbits; in addition cecum relative weight and cecum length were also recorded.

### Blood Hematology and Serum Metabolites

Blood samples from slaughtered rabbits were collected into sterile tubes. Blood parameters were measured (1, 13). To determine serum metabolites, the blood samples were left for clotting and centrifuged at 3,500 *rpm* × 15 min. The serum was then isolated and stored at −20°C until analysis. Serum metabolites were estimated using biochemical diagnostic kits and spectrometer (Shimadzu, Kyoto, Japan) (1, 14).

### Anti-oxidant Indices Assay in Serum

The serum samples were analyzed spectrophotometrically to determine the levels of reduced glutathione (GSH) activity (15) by using commercially purchased kits (Shimadzu, Kyoto, Japan). In addition to determining superoxide dismutase (SOD) activity (16) by kits (Biodiagnostic Company, El-Tahrir St. Dokki, Giza, Egypt).

### Cecal Microflora

Five rabbits were randomly selected from each treatment at 12 weeks of age and slaughtered by the same way as mentioned before, then three samples were taken from each rabbit as fresh digesta (1–2 g) from the middle part of the cecum and retained in bottles under a CO_2_ stream and rapidly transferred to the laboratory for determining bacterial populations (3). Microbial count was performed following the protocols of Xia et al. (17).

### Statistical Analysis

All data were analyzed using SAS (18) software. Data for growth performance, blood hematology and biochemistry, carcass properties and microbiological parameters were analyzed with one-way ANOVA by using the general liner model (GLM) according to the following model: X_ik_ = μ + T_*i*_ + e_ik_, where, X_*ik*_ = is the value of any observation, T_*i*_ = effect of treatment, BS at different levels (0, 0.25, 0.5, 0.75, 1 g/kg diet), e_ik =_ random error.

## Results

### Growth Performance

The findings of the growth performance criteria are illustrated in Table 2. The BS supplementation in rabbit diets at different levels (BS0.25, BS0.50, BS0.75, and BS1.0) not affects (*P* > 0.05) LBW at 0, 2, 4, and 6 weeks of feeding trial. The highest overall daily BWG (*P* < 0.05) was noted in BS0.50 supplemented rabbits compared to other groups. Rabbits that received BS0.25, BS0.50, and BS0.75diets consumed less feed and recorded the better FCR than others throughout the overall feeding period (0–6 weeks).

**Table 2:** Effect of *Boswellia serrata* on growth performance parameters of NZW rabbits.

**Items**	**Experimental groups (*****Boswellia serrata*** **%)**				
	**Control (0%)**	**BS 0.25%**	**BS 0.5%**	**BS 0.75%**	**BS 1.0%**
**Live body weight (g)**					
0 weeks	768.13 ± 75	791.25 ± 74	772.50 ± 74	799.38 ± 40	758.75 ± 55
2 weeks	1180.00 ± 105	1220.63 ± 96	1189.38 ± 112	1154.38 ± 92	1223.75 ± 159
4 weeks	1601.88 ± 148	1613.75 ± 100	1552.50 ± 128	1561.88 ± 164	1603.75 ± 197
6 weeks	1946.25 ± 124	1973.13 ± 112	1923.13 ± 148	2,025 ± 154	1979.38 ± 234
**Daily body weight gain (g)**					
0–2 weeks	30.00 ± 6.57^abc^	31.88 ± 6.11^ab^	35.80 ± 7.22^a^	26.14 ± 5.63^bc^	23.81 ± 4.69^c^
2–4 weeks	28.57 ± 3.54^ab^	32.77 ± 4.51^a^	30.27 ± 6.06^a^	28.39 ± 4.70^ab^	23.70 ± 3.94^b^
4–6 weeks	25.36 ± 3.78	25.18 ± 6.49	25.89 ± 7.56	26.43 ± 5.78	24.98 ± 5.15
Overall	27.98 ± 2.38^ab^	29.94 ± 4.15^ab^	30.65 ± 4.97^a^	26.99 ± 1.22^ab^	24.16 ± 0.71^b^
**Daily feed intake (g)**					
0–2 weeks	111.94 ± 7.77^a^	103.13 ± 7.50^b^	98.13 ± 16.24^bc^	94.22 ± 13.89^c^	103.97 ± 7.18^b^
2–4 weeks	128.57 ± 13.51^a^	105.16 ± 17.49^b^	104.38 ± 20.56^b^	108.44 ± 10.32^b^	104.38 ± 9.72^b^
4–6 weeks	137.86 ± 14.65^a^	114.84 ± 13.10^c^	132.19 ± 13.24^ab^	124.84 ± 12.47^bc^	121.25 ± 14.00^bc^
Overall	126.12 ± 13.13^a^	107.71 ± 6.26^b^	111.57 ± 18.13^b^	109.17 ± 25.83^b^	109.67 ± 4.94^ab^
**Feed conversion ratio (g/g)**					
0–2 weeks	3.88 ± 0.80^b^	2.86 ± 0.81^c^	3.31 ± 0.51^bc^	3.60 ± 0.63^b^	4.37 ± 1.07^a^
2–4 weeks	4.52 ± 0.27^b^	3.24 ± 0.52^c^	3.54 ± 0.91^c^	3.82 ± 0.91^b^	4.40 ± 0.77^a^
4–6 weeks	5.55 ± 1.02	4.89 ± 1.60	5.42 ± 1.30	4.72 ± 1.49	4.85 ± 0.78
Overall	4.65 ± 0.84^a^	3.81 ± 0.93^c^	3.94 ± 1.33^c^	4.05 ± 1.02^b^	4.54 ± 0.25^a^

*NZW, New Zealand White; BS0.25, basal diet + 0.25 g BS/kg diet; BS0.5, basal diet + 0.5 g BS/kg diet; BS0.75, basal diet + 0.75 g BS/kg diet; BS1.0, basal diet + 1 g BS/kg diet; SE, Standard error. Means in the same raw carrying different superscripts are statistically significant (P < 0.05)*.

### Carcass Measurements

The effects of dietary BS on the carcass traits and the cecum length and weight of rabbits are reported in Table 3. In overall, carcass parameters were not affected (*P* > 0.05) by BS supplementation, except for kidneys and heart weights. The BS1.0 supplemented rabbits showed significant decrease of kidney yield, but significant increase for relative heart weight compared to the control group.

**Table 3:** Effect of *Boswellia serrata* on carcass traits of NZW rabbits at 12 weeks of age.

**Items ** **(% on slaughter weight)**	**Experimental groups (*****Boswellia serrata*** **g/kg diet)**				
	**Control**	**BS 0.25**	**BS 0.5**	**BS 0.75**	**BS 1.0**
Carcass	56.42 ± 2.56	56.98 ± 4.79	56.41 ± 2.64	56.09 ± 2.73	55.94 ± 2.05
Liver	3.53 ± 0.43	3.55 ± 0.68	3.97 ± 0.32	3.51 ± 1.01	3.52 ± 0.55
Kidneys	0.64 ± 0.10^ab^	0.68 ± 0.03^ab^	0.72 ± 0.09^ab^	0.75 ± 0.09^a^	0.58 ± 0.07^b^
Lungs	0.49 ± 0.04	0.57 ± 0.06	0.58 ± 0.09	0.54 ± 0.02	0.58 ± 0.13
Heart	0.31± 0.04^b^	0.34 ± 0.08^ab^	0.32 ± 0.04^ab^	0.34 ± 0.02^ab^	0.41 ± 0.05^a^
Spleen	0.05 ± 0.02	0.05 ± 0.02	0.04 ± 0.02	0.05 ± 0.01	0.05 ± 0.01
Testis	0.23 ± 0.02	0.23 ± 0.02	0.21 ± 0.07	0.25 ± 0.05	0.25 ± 0.01
Gut	18.29 ±1.01	19.22 ± 3.74	17.33 ± 3.21	15.14 ± 2.12	17.71 ± 3.78
Caecum	0.37 ± 0.12	0.45 ± 0.10	0.44 ± 0.09	0.43 ± 0.14	0.44 ± 0.07
Appendix length (cm)	9.83 ± 1.26	11.37 ± 1.85	11.33 ± 076	11.17 ± 1.04	11.00 ± 1.00

*NZW, New Zealand White; BS0.25, basal diet + 0.25 g BS/kg diet; BS0.5, basal diet + 0.5 g BS/kg diet; BS0.75, basal diet + 0.75 g BS/kg diet; BS1.0, basal diet + 1 g BS/kg diet. Means in the same raw carrying different superscripts are statistically significant (P < 0.05)*.

### Blood Hematology

In the current study, all hematological parameters (erythrogram and leukogram) were not statistically influenced (*P* > 0.05) by the BS treatments except for MCV (Table 4). The BS supplementation at level BS0.5 significantly decreased the blood content of MCV compared to other treated and control groups. Furthermore, supplementing the diet with *B. serrata* (at BS0.5, BS0.75, BS1.0) enhanced total WBC and platelets in comparison with the control group.

**Table 4:** Blood parameters of growing NZW rabbits fed different levels of *Boswellia Sarrata* in diet at 12 weeks of age.

**Items**	**Experimental groups (*****Boswellia serrata*** **g/kg diet)**				
	**Control**	**BS 0.25**	**BS 0.5**	**BS 0.75**	**BS 1.0**
**Erythrogram**					
RBC (10^6^/mm^3^)	5.79 ± 0.82	5.31 ± 0.10	5.16 ± 0.40	5.42 ± 0.44	5.63 ± 0.63
HGB(g/dL)	12.30 ± 1.15	12.23 ± 0.64	11.60 ± 0.78	12.57 ± 0.64	12.53 ± 1.41
MCHC (pg)	33.56 ± 0.59	34.97 ± 1.19	34.40 ± 1.15	3.92 ± 1.13	34.55 ± 2.13
MCV(μm^3^)	63.76 ± 2.92^ab^	66.02 ± 2.19^a^	59.43 ± 2.86^b^	66.52 ± 1.46^a^	64.46 ± 1.65^ab^
HCT (%)	36.79 ± 3.94	35.06 ± 1.33	33.75 ± 2.70	36.02 ± 2.39	36.33 ± 4.33
PLT (10^3^/μL)	271.33 ± 81	265.67 ± 11	365.00 ± 132	382.00 ± 98	405.67 ± 122
**Leukogram**					
WBC (10^3^/mm^3^)	5.65 ± 1.93	5.68 ± 2.45	7.52 ± 1.76	5.23 ± 0.14	6.86 ± 1.47
LYM (10^3^/mm^3^)	66.81 ± 3.50	69.12 ± 18.20	59.75 ± 12.61	64.16 ± 5.67	59.47 ± 4.66
MID (10^3^/mm^3^)	12.83 ± 2.91	12.56 ± 7.78	11.25 ± 4.67	11.30 ± 1.67	12.87 ± 4.75
GRA (10^3^/mm^3^)	20.36 ± 0.70	18.33 ± 10.61	29.00 ± 13.87	24.54 ± 4.36	27.66 ± 3.77

*NZW, New Zealand White; BS0.25, basal diet + 0.25 g BS/kg diet; BS0.5, basal diet + 0.5 g BS/kg diet; BS0.75, basal diet + 0.75 g BS/kg diet; BS1.0, basal diet + 1 g BS/kg diet; RBCs, red blood cells; HGB, hemoglobin; MCHC, Mean corpuscular hemoglobin count; MCV, mean corpuscular volume; HCT, haematocrit; PLT, platelet count; WBCs, white blood cells; LYM, lymphocytes; MID, mid-range; GRA, granulocytes. Means in the same raw carrying different superscript letters are statistically significant (P < 0.05)*.

### Serum Biochemistry

The effects of dietary inclusion of BS on serum metabolites of animals are revealed in Table 5. No difference was detected in any of serum metabolites parameters except for albumin, A/G ratio, TC, TG, LDL, and ALT. The BS supplementation (BS0.75 and BS1.0) reduced (*P* < 0.01) blood level of albumin compared to control. Moreover, BS0.25, BS0.75, and BS1.0 supplemented rabbits showed decreased (*P* < 0.05) A/G ratio compared to control group. Feeding of 1.0 g BS/kg produced lower values of TC than that of the other treatments. A significant (*P* < 0.05) was observed in the serum level of both TG and LDL as a result of supplementing rabbit diet with BS (BS0.25, BS0.5, BS0.75, and BS1.0), compared to control diet. For liver function, ALT activity was increased (*P* < 0.01) due to dietary treatments at level BS0.25 when compared to control group. Additionally, the effect of dietary BS on anti-oxidant status of growing rabbits is reported in Table 5. All the aforementioned patterns were insignificantly affected by enriching rabbit diets with different levels of BS.

**Table 5:** Effects of *Boswellia serrata* on serum metabolites of NZW rabbits.

**Items**	**Experimental groups (*****Boswellia serrata*** **%)**				
	**Control (0%)**	**BS 0.25%**	**BS 0.5%**	**BS 0.75%**	**BS 1.0%**
Total protein (g/dL)	5.40 ± 0.12	5.56 ± 0.39	5.61 ± 0.82	4.83 ± 0.83	5.07 ± 0.78
Albumin (g/dL)	3.76 ± 0.10^a^	3.34 ± 0.43^ab^	3.50 ± 0.74^ab^	2.82 ± 0.21^b^	2.73 ± 0.37^b^
Globulin (g/dL)	1.64 ± 0.06	2.31 ± 0.75	2.10 ± 0.08	2.01 ± 0.19	2.34 ± 0.41
A/G ratio	2.29 ± 0.11^a^	1.60 ± 0.75^b^	1.66± 0.25^ab^	2.34± 0.41^b^	1.17 ± 0.05^b^
Creatinine (mg/dL)	0.72 ± 0.12	0.82 ± 0.18	0.75 ± 0.18	0.75 ± 0.14	0.83 ± 0.16
Urea (mg/dL)	25.73 ± 8.35	26.33 ± 5.89	23.60 ± 6.81	23.25 ± 4.53	18.99 ± 3.69
TC (mg/dL)	102.40 ±12.22^a^	73.04 ±11.35^ab^	91.16 ± 9.74^ab^	83.41 ± 12.81^ab^	64.29 ± 9.63^b^
TG (mg/dL)	161.78± 10.05^a^	101.65 ±14.52^b^	84.09 ± 10.20^b^	101.99 ± 11.34^b^	94.11± 10.07^b^
HDL (mg/dL)	55.93 ± 5.45	40.69 ± 10.74	38.82 ± 21.07	34.37 ± 6.02	39.38 ± 8.30
LDL (mg/dL)	22.21 ± 2.34^a^	16.21 ± 1.38^b^	15.44 ± 3.70^b^	12.35± 1.91^b^	14.42 ± 2.61^b^
Glucose	162.55 ± 37.35	183.16 ± 37.53	160.39 ± 4.42	178.23 ± 11.08	194.24 ± 17.82
AST (U/L)	70.73 ± 14.92	54.56 ± 3.53	63.53 ± 14.70	59.52 ± 14.50	67.39 ± 13.90
ALT (U/L)	29.64 ± 1.76^b^	44.85 ± 3.16^a^	35.02 ± 2.21^ab^	41.23 ± 3.46^ab^	38.95 ± 3.36^ab^
SOD (U/mL)	0.15 ± 0.50	0.12 ± 0.04	0.18 ± 0.02	0.17 ± 0.04	0.19 ± 0.04
GSH (mg/mL)	0.11 ± 0.02	0.15 ± 0.02	0.19 ± 0.09	0.16 ± 0.03	0.18 ± 0.05

*BS0.25, basal diet + 0.25 g BS/kg diet; BS0.5, basal diet + 0.5 g BS/kg diet; BS0.75, basal diet + 0.75 g BS/kg diet; BS1.0, basal diet + 1 g BS/kg diet; A/G, albumin/ globulin ratio; TC, total cholesterol; TG, triglycerides; HDL, high density lipoprotein; LDL, low density lipoprotein; AST, aspartate aminotransferase; ALT, alanine aminotransferase; SOD, superoxide dismutase; GSH, glutathione. Means in the same raw carrying different superscripts are statistically significant (P < 0.05)*.

### Cecal Microbiota

Dietary BS supplementation led to significant changes of caecal content of microbial populations (*P* < 0.05) in growing rabbits (Table 6). It was found that feeding of BS at 0.25, 0.50, 0.75, or 1.0 g/kg diet resulted in a significant (*P* < 0.05) suppression of total bacterial count (TBC) and *Salmonella enteritidis* PT4 compared to control rabbit group; in addition, rabbits receiving BS at 0.25, 0.50, and 1.0 g/kg showed the highest decrease of *Escherichia coli* compared to control treatment.

**Table 6:** Effects of *Boswellia serrata* on cecal microbiota (total bacterial count, *Escherichia coli*, and *Salmonella enteritidis*) in NZW growing rabbits.

**Items**	**Experimental groups (*****Boswellia serrata*** **%)**				
	**Control (0%)**	**BS 0.25%**	**BS 0.5%**	**BS 0.75%**	**BS 1.0%**
Total bacterial count	188.12 ± 12.73^a^	58.95 ± 9.24^b^	33.41 ± 2.41^b^	48.76 ± 10.15^b^	45.13 ± 6.18^b^
*Escherichia coli* ATCC 8739	52.89 ± 3.033^a^	36.15 ± 3.18^b^	14.33 ± 3.25^c^	46.28 ± 3.81^ab^	37.95± 5.35^b^
*Salmonella enteritidis* PT4	57.42 ± 6.65^a^	21.35 ± 4.65^bc^	26.47 ± 6.14^b^	14.65 ± 5.29^c^	21.17 ± 3.78^bc^

*NZW, New Zealand White; BS0.25, basal diet + 0.25 g BS/kg diet; BS0.5, basal diet + 0.5 g BS/kg diet; BS0.75, basal diet + 0.75 g BS/kg diet; BS1.0, basal diet + 1 g BS/kg diet. Means in the same raw carrying different superscripts are statistically significant (P < 0.05)*.

## Discussion

The therapeutic activity of *B. serrata* has been previously reported by several authors (19, 20). The *B. serrata* essential oil has plentiful attributes of interest, comprising anti-bacterial, anti-fungal, anti-inflammatory, anti-tumor, and anti-oxidant activities as well as immunostimulatory agent, and it was successfully used as natural feed additive for poultry (5, 6, 8, 9, 20); however, there is a lack of information on the use of this medicinal plants in rabbit.

Generally, medicinal plants and their secondary metabolites have been used for improving the efficiency and productivity of livestock production (1, 21). The present study showed beneficial impacts of dietary BS supplementation on the LBW and BWG of growing rabbits. The BS has beneficial therapeutic features in abundant pathological situations favored by natural medicine scientists in European countries, because of its possible nutritional and medical applications. It reveals an assortment of beneficial properties, which is related to the many aromatic compounds such as boswellic acid (7, 8).

In the present trial, live body weight and gain for NZW rabbits receiving BS diets were higher compared to unsupplemented group. The use of a 0.25–0.75 g/kg diet of BS the rabbit diets enhanced the feed consumption and efficiency. Previous studies have been shown the similar positive impact of phytogenic feed additives in animal production (1, 4, 8, 9, 21). The enhancement in LBW and BWG by BS, as a natural growth promoter, may be attributed to the presence of some active compounds (such as boswellic acid) which stimulates the ability to absorb and digest nutrients (5–9). Our findings revealed that dietary supplementation with BS significantly increased LBW compared with control (Table 2). At the same manner, Al-Yasiry et al. (8) hypothesized that the addition of *B. serrata* resin (3, 4, or 5%) to broiler diets had a positive effect on chicken growth performance; this is consistent with previous studies by Kiczorowska et al. (5). It was also confirmed (9) that the supplementation of 2.0–2.5% BS to broiler diets improved growth performance compared to unsupplemented group, indicating that high levels of BS may be desirable as an additive to feed to achieve growth enhancement features. The BS effect is associated with high absorption, which promotes the release of digestive enzymes which increases performance, intestinal villi morphology and also declines the velocity of material transit (22). This positive consequence of BS on gut was previously observed (5, 6). Normal morphology and functions of gut are also related to an improved nutrient availability, as demonstrated by the enhancement of organic matter digestibility of diet (8). However, the BS levels above 4% showed the deleterious properties of the phytogenic feed additives, and this was found by Singh et al. (23) using rats.

As can be seen from Table 3, feeding of BS showed no significant effect on the percentages of most of the carcass traits and edible organs, these findings are in line with those of previous studies suggesting that, phytogenic feed additives (as coconut oil, thyme, eucalyptus, cinnamon*, Melaleuca alternifolia, Echinacea angustifolia*, pepper and ginger extracts) has no influence on carcass traits and relative size of edible organs when added to diet (1, 24). We only observed an enhancement of the heat and kidney weight by BS supplementation to a growing rabbit diet. In contrast with our findings, Al-Yasiry et al. (9) concluded that the best-quality carcass with a high proportion of muscles and low fat content (control vs. BS diets, at *P* < 0.05) was obtained in broiler chickens fed diets supplemented with 2.0–2.5% of *B. serrata*.

An important variable in the evaluation of the efficacy of BS diets could be the course of metabolic processes, such as the alterations of blood parameters of growing rabbit (24). The hematological and biochemical parameters in the blood are reflecting health status of animals, so in our study, it was shown that these values were within the normal values, without toxicity signs. The supplementation with BS decreased significantly the blood content of albumin, total cholesterol, triglycerides, and LDL compared to no additive group. Also, we observed an increase in liver function (ALT) in rabbits fed BS than the control group. Plasma proteins are good variables screening the nutritional and health status of the organism, which have multifunction in the body such as enzymatic, transport, and immune functions (8). Our findings showed that a significant increase in the albumin content in rabbits fed BS diet. Higher serum globulin level is a valuable index of immunological response and production of anti-bodies (8, 25). Additionally, it has been observed that supplementation with 0.25, 0.50, or 0.75 g/kg *B. serrata* significantly decreased total cholesterol level, triglycerides, and LDL in the blood serum in growing rabbits (Table 4). In agreement with our findings, it was found (1, 2) that supplementation of diet with phytogenic compounds reduced blood lipids. Here we provide evidence that the interpretation of this finding can be achieved by a reasonable reduction in the synthesis of acetyl CoA enzyme which is necessary for fatty acids biosynthesis in mitochondria. Moreover, the supplementation of herbs or spices in the diet of rabbits could support enzymes activity involved in the cholesterol conversion to bile acids, and then led to a decrease of cholesterol level in rabbit carcasses (1). The liver serves a main function in the body, so the assessment of liver enzymes activities is an efficient index of normal health status of growing rabbit fed BS. In accordance with the result of this study, it was suggested that the rapid metabolization of secondary metabolites of medicinal plants in the liver may cause an impairment of its function therefore, increase enzyme levels (AST and ALT) from the liver (1). Conversely, a significant decrease in liver enzymes activity within the normal values in *B. serrata* supplemented diet to broilers at 3 to 5% level (8). Likewise, the findings achieved by Aliyu et al. (26) stated that the *Boswellia dalzielii* extract reduced the activity of serum AST in treated rats, compared to control. The hepatoprotective effect of this plant was due to f natural flavonoids and polyphenols, possessing protective activity (5, 6, 8, 27). This consequence is in line with that described by Ibrahim et al. (28), who found a defensive effect of in rats with liver damage induced by paracetamol demonstrating the inhibitory effect of BS extracts on 5-lipoxygenase synthesis, being responsible of the hepatocytes inflammation (1, 8).

Giving the influence of BS on the microbial population in the rabbit cecum, in the present study, dietary BS resulted in a respite in the spoilage occurrence. In addition, 0.25 to 0.75 g/diet in diet repressed the growth of total bacterial count, *E. coli* and *S. enteritidis* spp. in rabbit cecum, and this could be due to the presence of Boswellic acid which present as the powerful anti-microbial effect and also, due to the high polyphenols in BS (5, 6, 19). Similar *in vitro* observation can be made by Chevrier et al. (29) in terms; *Boswellia carterii* extract inhibits TH1 cytokines and promotes TH2 cytokines. The anti-microbial activity of plant extracts or its secondary metabolites is faithfully associated with their polyphenols content reflecting the effective bioactive compounds having anti-oxidant and anti-microbial actions (2). Polyphenols may modify the pH gradient and the membrane of bacterial cells potential leading to disruption of the intracellular ATP content of *Salmonella* and *E. coli* (3). Nowadays, medicinal plants are widely used around the world to inhibit the growth of Gram-negative bacteria, as an alternative to pharmaceutical drugs. The anti-bacterial resin properties may be attributed to the presence of phenolic acid in boswellic acid. Phenolic compounds in organic acids improve the efficacy of protein and energy by reducing microbial competition with the host for nutrients and endogenous nitrogen losses, by lowering the incidence of subclinical infections and secretion of immune mediators, and by reducing the production of ammonia (29–31).

## Conclusions

Our results concluded that dietary *B. serrata* supplementation up to 0.75 g/kg diet to rabbit feed has the ability to enhance the FCR. Rabbits fed a diet an enriched with BS up to 1.0 g/kg diet had decreased blood lipid profile and the lower counts of total bacterial count, *Escherichia coli*, and *S. enteritidis* in the cecum content. So, it could be recommended to reinforce the diets of growing rabbits with BS to enhance growth performance, microbiological status of the cecum, and blood lipid profile. Thus, *B. serrata* can be considered as a safe and effective dietary natural feed additive to the diet of growing rabbits.

## Data Availability Statement

All of the data produced and analyzed in the present study are involved in the manuscript as tables. The corresponding author will respond to the requests concerning the raw data and reasonable accommodations will be provided.

## Ethics Statement

The animal study was reviewed and approved by Department of Poultry, Faculty of Agriculture, Zagazig University, Egypt.

## Author Contributions

All authors listed have made a substantial, direct and intellectual contribution to the work, and approved it for publication.

### Conflict of Interest

The authors declare that the research was conducted in the absence of any commercial or financial relationships that could be construed as a potential conflict of interest.
